# COVID-19 in Patients with Active Tuberculosis

**DOI:** 10.3390/diagnostics11101768

**Published:** 2021-09-26

**Authors:** Monika Kozińska, Ewa Augustynowicz-Kopeć

**Affiliations:** Department of Microbiology, National Tuberculosis and Lung Diseases Research Institute, Plocka 26, 01-138 Warsaw, Poland; e.kopec@igichp.edu.pl

**Keywords:** COVID-19, tuberculosis, SARS-CoV-2, *Mycobacterium tuberculosis*, coincidence, coinfection

## Abstract

Data on the coincidence of tuberculosis (TB) and COVID-19 are limited, and previous observations are based on the results of just a few studies, which has led to polarized views on the course of infection with SARS-CoV-2 in patients with active TB. We present the first two cases of TB and COVID-19 coinfection in the population of patients in Poland, diagnosed shortly after the outbreak of the global pandemic. In the first patient, TB was very advanced at the time of infection with SARS-CoV-2. From the third day of hospitalisation, respiratory failure was increasing, with no improvement after the use of high-flow oxygen therapy and mechanical ventilation. On the seventh day of hospitalization, the patient died. In the second presented case, therapeutic success was achieved despite the coincidence of COVID-19, infection with HIV, and extrapulmonary and pulmonary TB. The patient had symptoms of renal failure and the SARS-CoV-2 infection was mild and asymptomatic. Because both patients were in the care of a homeless shelter, a molecular epidemiological investigation was carried out. Different DNA profiles of *Mycobacterium tuberculosis* complex isolates detected in clinical materials from patients ruled out the transmission of tuberculosis. Based on our analysis, it is impossible to clearly define the influence of active TB on the course of SARS-CoV-2 infection. We can only suggest that coinfection is particularly dangerous for socially disadvantaged people, the elderly, and people with other comorbidities. In the coming years, a negative impact of the current pandemic on control programmes will be observed for many infectious diseases, including TB.

## 1. Introduction

The global risks of infectious diseases, including tuberculosis, have long been a concern of governmental and non-governmental institutions responsible for public health policy. Since tuberculosis as an infectious disease remains a major cause of death in the world, it requires monitoring, efficient and reliable diagnosis, contact tracing, and effective treatment. The COVID-19 pandemic has significantly changed the functioning of healthcare systems. COVID-19 and tuberculosis are both infectious diseases that primarily affect the respiratory system and cause similar symptoms, such as cough, fever, and breathing problems [1]. Both diseases are transmitted mainly by close contact and usually spread via airborne droplets. Factors influencing the course of COVID-19 include old age [2], immune response [3,4], host genetics [5], environmental factors [6], and therapeutic interventions related to comorbidities [7,8]. Mass immunization programmes in populations all over the world are now in progress, and treatment largely relies on adjuvant therapy [9].

The incidence of tuberculosis in Poland is slightly higher than the European average, and in 2019 it was 13.9/100,000. Poland, Romania, and the United Kingdom accounted for 45% of all TB cases reported in the European Union [10]. Considering the number of SARS-CoV-2 cases, Poland, until October 2020, was not a high-risk country compared to European data. However, as the pandemic continued, the domestic epidemiological situation got worse, and the notified statistics reached alarming levels. According to the ECDC update (as of 21 July 2021), 2,881,594 cases of SARS-CoV-2—including 75,219 deaths (2.6% of infected patients)—have been reported in total from Poland [11].

In the face of the global COVID-19 pandemic, other epidemiological problems important for public health have been neglected, and there have been shortfalls in the diagnosis of many serious infectious diseases, including TB.

The National Reference Laboratory for Tuberculosis (NRLT) at the Institute of Tuberculosis and Lung Diseases in Warsaw has monitored the incidence of TB and COVID-19 coinfection in Poland from the beginning of the pandemic and holds relevant records of patients. Based on notifications from regional laboratories for *Mycobacterium tuberculosis*, two cases of TB and COVID-19 coinfection have been documented, and they are described below.

## 2. Case Study

### 2.1. Case 1—Lethal COVID-19 and TB Infection

A 71-year-old homeless man reported to the emergency department on 25 May 2020 due to general weakening, cough with expectoration of greenish sputum of over a week, and atrial fibrillation of undetermined duration. He had a fever of 39.6 °C and a sore throat. His general condition was moderately severe; he was conscious, in logical contact, cachectic (body mass index: 15.59), and with severe dyspnoea. Vital signs were an average heart rate of 128 beats per minute, blood pressure 91/67 mmHg, tachypnea, with a respiratory rate of 44 breaths/min, oxygen saturation (spO2) on room air 76–89%, with an increase in spO2 up to 96% after oxygen therapy through a mask with a flow of 5 Lt/min. The medical history revealed smoking (more than 20 cigarettes/day) for most of his life. The patient was treated for tuberculosis in 1981.

**Figure 1:**
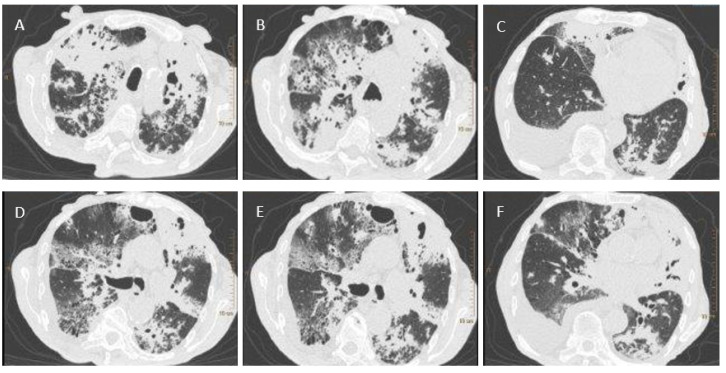
Figure **1.** Chest CT scans. (**A**)—parenchymal-atelectatic lesions; thick-walled cavities filled with masses of tissue decay in the upper lobes; (**B**)—parenchymal-atelectatic lesions; cavities filled with tissue decay in segment 3 of the right and left lung, with the accompanying areas of tree-in-bud-appearance in segment 3 of the left lung and in the apex of segment 6 of the right and left lung; (**C**)—fluid in the pleural cavity; (**D**–**F**)—tree-in-bud-appearance in the apex of segment 6 of the right lung and in segments 6–9 of the left lung.

A test for SARS-CoV-2 was performed using a throat and nasal swab taken on 27 May 2020. The Vitassay qPCR SARS-CoV-2 test was positive for SARS-CoV-2 RNA. A chest X-ray revealed bilateral areas of confluent parenchymal and interstitial densities in the lungs, predominantly in the upper and middle fields. Computed tomography, completed on 28 May 2020, showed lesions typical of pulmonary tuberculosis (Figure 1). Antimycobacterial treatment was initiated empirically in the following scheme: isoniazid (INH), rifampicin (RMP), pyrazinamide (PZA), and ethambutol (EMB).

**Figure 2:**
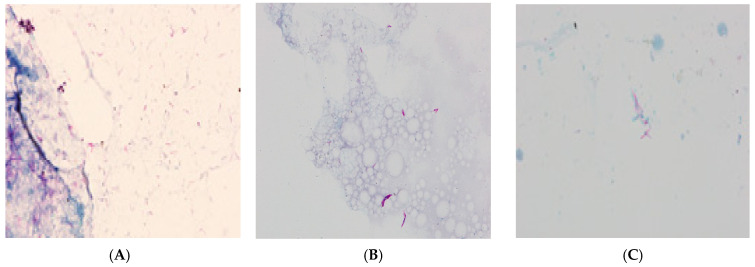
Acid-fast bacilli visible after Ziehl–Neelsen staining; (**A**)—sputum (Patient 1); (**B**)—urine (Patient 2); (**C**)—sputum (Patient 2).

For further TB diagnosis, sputum was sampled from the patient and sent to the NRLT. The analysis of the fluorescent-stained microscopic smear using the Ziehl–Neelsen technique revealed the presence of acid-fast bacilli (AFB+++) (Figure 2A). The GeneXpert assay confirmed the presence of genetic material from MTBC. An identification and drug sensitivity test for the isolated strain confirmed it belonged to the species *Mycobacterium tuberculosis* and was sensitive to streptomycin (SM), INH, RMP, EMB, and PZA.

From the third day of hospitalisation, respiratory failure was increasing, with no improvement after the use of high-flow oxygen therapy (helmet-based CPAP/continuous positive airway pressure). Mechanical ventilation was applied, and an infusion of norepinephrine and dobutamine was initiated due to circulatory decompensation. Cardiac arrest by asystole occurred after a few hours and the patient was pronounced dead.

### 2.2. Case 2—Asymptomatic COVID-19 in the Coexistence of Extra-Pulmonary and Pulmonary TB and HIV Infection

An HIV-positive, 64-old patient was treated in 2018–2019 for pulmonary tuberculosis and urogenital tuberculosis, which was confirmed by a positive bacteriological test in NRLT. Back then, assays revealed the presence of mycobacteria in sputum and urine smears (Figure 2B,C). In the genetic assay, *Mycobacterium tuberculosis* complex (MTBC) DNA was detected in clinical materials, and mycobacterial strains sensitive to SM, INH, RMP, EMB, and PZA were cultured.

From 27 May 2020, the patient was hospitalized due to haematuria and chronic kidney disease requiring dialysis. Relevant diagnostic procedures were carried out because of the suspected relapse of urogenital TB. Histopathological examination of bladder specimens (collected on 28 May) revealed granular lesions with purulent inflammation and necrotizing purulent masses of tissue. On 19 June 2020, tissue sections fixed in paraffin were submitted to the NRLT for TB diagnosis. The genetic assay was negative for MTBC DNA.

During hospitalization, the patient tested positive for SARS-CoV-2. The analytical material was a throat and nasal swab taken on 27 May 2020. The Vitassay qPCR SARS-CoV-2 test was positive for SARS-CoV-2 RNA. Due to mild symptoms of infection, the patient did not require clinical intervention and was discharged from the hospital in good health. He received outpatient care, with dialysis therapy recommended.

### 2.3. Molecular Epidemiological Investigation

An interview revealed that both patients were in the care of a homeless shelter in Warsaw and had contact with each other. A molecular epidemiological investigation was carried out with a focus on the potential transmission of TB between patients. MTBC strains isolated from clinical samples from both patients were analysed using spoligotyping and MIRU-VNTR. The strain cultured from Patient 1′s sputum was classified as the spoligotype LAM9 42 with MIRU-VNTR code 233464236654438. Strains cultured from the sputum and urine of Patient 2 were identical and represented the molecular family T1 51 with MIRU-VNTR code 324443322564234 (Table 1).

## 3. Discussion

Data on the coincidence of TB and COVID-19 are limited. So far, there have been a few studies describing cases of coinfection with MTBC and the SARS-CoV-2 virus [12,13,14,15]. In our research, we present the first two cases of coinfection in Poland, detected shortly after the outbreak of the global pandemic. Both of the case study patients had active TB and contracted a SARS-CoV-2 infection. However, the course of coinfection was entirely different in the first patient, and at the time of infection with the virus, the TB was very advanced. This caused worse outcomes, and the patient died as a result of both diseases. The second presented case is particularly interesting because therapeutic success was achieved despite the coincidence of COVID-19, HIV, and extrapulmonary and pulmonary TB. Hypothetically, this homeless patient, because of coinfection with TB and HIV, had better access to healthcare, and the treatment of TB did not cause damage and complications as in the first patient.

Polarized views have been reported regarding the course of SARS-CoV-2 infection in patients with active TB. The first cohort analysis to assess the relationship between TB and COVID-19 was prepared through an international collaboration and included 49 cases of coinfection identified in 8 countries and revealed a higher mortality rate among the elderly with a history of tuberculosis [13]. Chen et al. reported that tuberculosis increased susceptibility to COVID-19 and exacerbated its symptoms [16]. Similarly, Italian researchers have suggested that coinfection may be more severe in the elderly or in patients with comorbidities, but that it is a clinically manageable condition [17,18]. Another study carried out in the Philippines confirmed a negative role of TB in the course of COVID-19, and linked coinfection with a higher risk of morbidity and mortality [19]. Substantial evidence for the impact of TB on COVID-19 outcomes was obtained in a South African study that compared data on more than 3 million patients treated in public healthcare institutions, with or without COVID-19, and with other comorbidities, including TB and HIV. It demonstrated that a history of tuberculosis, active tuberculosis, and tuberculosis coexisting with HIV infection all increase the risk of death in COVID-19 patients [20].

Contrasting conclusions were reached in another two studies, which found no direct relationship between tuberculosis and the deterioration of COVID-19 symptoms [21,22].

## 4. Conclusions

Perhaps it is too early and too small a population has been studied to draw firm conclusions on how TB affects the course of SARS-CoV-2 infection. Over time, more studies will probably be published to support these hypotheses. By consensus, it can already be inferred that coinfection is particularly dangerous for socially disadvantaged people, the elderly, and patients with other comorbidities such as diabetes and/or hypertension. However, taking into account the cases reported by us, it may not be possible to draw firm conclusions on the consequences of viral and bacterial coinfections.

The consequences of the COVID-19 pandemic pose a serious challenge to tuberculosis control programmes, mainly because of shortfalls in the diagnosis and treatment of tuberculosis [23]. Due to the similarities between the symptoms of TB and COVID-19, countries without an effective diagnostic framework are unable to correctly detect these infections. This issue has a negative effect on therapeutic decisions and, thus, on the prognosis of both diseases [24,25,26].

Based on these observations, it can be predicted that the SARS-CoV-2 pandemic is likely to impact healthcare systems around the world. Deficiencies in control programs for many chronic diseases (such as diabetes, COPD, hypertension, asthma, cardiovascular disease, cancer, and depression), diagnostic delays, and drug unavailability worsened the epidemiological situation and increased the number of cases and deaths [27]. In addition, lockdowns and increased difficulty in accessing healthcare resulted in the adoption of virtualized treatments that eliminated the possibility of physical meetings between patients and healthcare providers [28].

According to the WHO forecast, the SARS-CoV-2 pandemic will set the world programme of tuberculosis control back 5–8 years. In view of this situation, an increase in TB cases and deaths back to the level of the epidemiological indicators in 2013–2016 is expected in 2021.

It is essential for policymakers and financing institutions to recognize the seriousness of this problem and to take actions to enable the rapid implementation of innovative people-centred approaches to patients affected by TB so that the fight to end one pandemic does not aggravate another.

## Figures and Tables

**Table 1:** Clinical and microbiological characteristics of reported cases.

	PATIENT 1 Hospitalisation 25–31 May 2020	PATIENT 2 Hospitalisation 27.05–29 May 2020
Sex	Male	Male
Age	71	64
Social status	Homeless	Homeless
Comorbidities	Atrial fibrillation	HIV-positive Renal insufficiency
Symptoms on admission	Cough, weakness, malaise, coughing up sputum	No data
**TB**		
Medical history	1981—pulmonary tuberculosis	December 2018—pulmonary tuberculosis February 2019—urogenital tuberculosis
Chest X-ray, CT	(28 May 2020) Lesions suggesting pulmonary TB	No data
Bacteriological assay	(29 May 2020) Sputum AFB (+++)—MTBC culture	Sputum AFB (+)—MTBC culture Urine AFB (+++)—MTBC culture
Genetic assay	(29 May 2020) GeneXpert Sputum (+)	(19 June 2020) GeneXpert Bladder specimens embedded in paraffin (-)
Strain drug sensitivity	Sensitive to SM, INH, RMP, EMB, PZA	Sensitive to SM, INH, RMP, EMB, PZA
Strains genotyping Spoligotyping MIRU-VNTR	■■■■■■■■■■■■■■■■■■■■□□□□■■■■■■■■□□□□■■■■■■■ LAM9 42 233464236654438	■■■■■■■■■■■■■■■■■■■■■■■■■■■■■■■■□□□□■■■■■■■ T1 51 324443322564234
TB treatment	INH+RMP+PZA+EMB	No data
**COVID-19**		
Diagnostic tests	Throat and nasal swab	Throat and nasal swab
Genetic assay	Vitassay qPCR SARS-CoV-2 (+) (27 May 2020)	Vitassay qPCR SARS-CoV-2 (+) (27 May 2020)
Clinical symptoms	Cough, fever of 39.6 °C, shortness of breath, and a sore throat	No symptoms
Radiological observations	Chest X-ray: bilateral areas of fusing parenchymal-atelectatic densities in the lungs, predominant in the upper and middle fields CT scans: Extensive lesions parenchymal-atelectatic; thick-walled cavities filled with masses of tissue decay in the upper lobes płuc; areas of tree-in-bud-appearance in segment 3 of the left lung and in the apex of segment 6 of the right and left lung; fluid in the pleural cavity; tree-in-bud-appearance in the apex of segment 6 of the right lung and in segments 6–9 of the left lung	No data
Other diagnostic tests	-	Histopathological examination of bladder specimens revealed granular lesions with purulent inflammation and necrotizing purulent masses of tissue
**OUTCOME**	Unsuccessful treatment, death (31 May 2020)	Successful treatment, outpatient care

## Data Availability

The clinical data of the reported results have been archived in IV-th Department, Hospital for Infectious Diseases and in Department of Diagnostic Imaging, Hospital for Infectious Diseases. Microbiology data has been archived at the Department of Microbiology, National Tuberculosis and Lung Diseases Research Institute, Warsaw, Poland.
